# SDF-1α upregulation by atorvastatin in rats with acute myocardial infarction via nitric oxide production confers anti-inflammatory and anti-apoptotic effects

**DOI:** 10.1186/1423-0127-19-99

**Published:** 2012-11-21

**Authors:** Ruofeng Qiu, Anping Cai, Yugang Dong, Yingling Zhou, Danqing Yu, Yuli Huang, Dongdan Zheng, Shaoqi Rao, Yingqing Feng, Weiyi Mai

**Affiliations:** 1 Department of Cardiology, The First Affiliated Hospital of Sun Yat-sen University, 58 Zhongshan Road 2, Guangzhou, 510080, China; 2 Guangdong Provincial Cardiovascular Institute, Guangdong Provincial People’s Hospital, Guangdong Academy of Medical Sciences, Guangzhou, 510080, China; 3 Department of Epidemiology and Health Statistics, Sun Yat-sen University, Guangzhou, 510080, China

**Keywords:** Acute myocardial infarction, Atorvastatin, Stromal cell derived factor-1alpha

## Abstract

**Background:**

The effects of atorvastatin on SDF-1α expression under acute myocardial infarction (AMI) are still unclear. Therefore, our present study is to investigate the roles and mechanisms of atorvastatin treatment on SDF-1α expression in rats with AMI.

**Methods:**

Male Sprague–Dawley rats were underwent permanent coronary artery ligation and randomly assigned into four groups as follow: blank control (B), atorvastatin (A), atorvastatin plus L-NAME (A+L-NAME), and atorvastatin plus AMD3100 (A+AMD3100). Rats underwent similar procedure but without ligation were used as group sham operated (S). Atorvastatin (10mg/Kg/d body weight) was administrated by gavage to rats in three atorvastatin treated groups, and L-NAME (40mg/Kg/d body weight) or AMD3100 (5mg/Kg/d body weight) was given to group A+L-NAME or A+AMD3100, respectively.

**Results:**

Comparing with group B, NO production, SDF-1α and CXCR4 expression were significantly up-regulated in three atorvastatin treated groups at the seventh day. However, the increments of SDF-1α and CXCR4 expression in group A+L-NAME were reduced when NO production was inhibited by L-NAME. Anti-inflammatory and anti-apoptotic effects of atorvastatin were offset either by decrease of SDF-1α and CXCR4 expression (by L-NAME) or blockage of SDF-1α coupling with CXCR4 (by AMD3100). Expression of STAT3, a cardioprotective factor mediating SDF-1α/CXCR4 axis induced cardiac protection, was up-regulated most significantly in group A. The effects of atorvastatin therapy on cardiac function were also abrogated either when SDF-1α and CXCR4 expression was diminished or the coupling of SDF-1α with CXCR4 was blocked.

**Conclusion:**

SDF-1α upregulation by atorvastatin in rats with AMI was, at least partially, via the eNOS/NO dependent pathway, and SDF-1α upregulation and SDF-1α coupling with CXCR4 conferred anti-inflammatory and anti-apoptotic effects under AMI setting which we speculated that ultimately contributed to cardiac function improvement.

## Background

SDF-1α (stromal cell-derived factor-1alpha) is a small cytokine belonging to the chemokine family that is officially designated as Chemokine (C-X-C motif) ligand 12 (CXCL12). CXCR4 is a receptor for the C-X-C chemokine CXCL12/SDF-1 that transduces a signal by increasing intracellular calcium ions levels. Accordingly, SDF-1α constitutively expresses in cardiac tissues and correspondingly increases after acute myocardial infarction (AMI) so as to conduct self-protective process [1-3]. Through the coupling with its cognate receptor CXCR4, SDF-1α exerts multiple protective effects, as anti-apoptosis, pro-survival, cardiac remodeling amelioration, endothelial progenitor cells recruitment and neo-vascularization, to improve cardiac function [4-7]. However, the natural physiologic reaction of transient and slight upregulation of SDF-1α after AMI is far from sufficient to fully restore cardiac function [1,8,9]. Therefore, in animal models of ischemic cardiac diseases, many genetically manipulative strategies aiming to extending and enhancing SDF-1α expression have been intensively investigated [9-11]. The outcomes are striking and shed promising lights on SDF-1α potentially therapeutic efficacy on ischemic diseases. Notwithstanding, those novel genetic approaches seem infeasible in clinical practice at present time. Hence, it is warranted and necessitated to investigate whether commonly used medications have potential to up-regulate SDF-1α expression after AMI. To our knowledge, there has been rare study to explore this kind of effect with commonly used medications. Previously, Xu and colleagues reported that SDF-1α expression in ischemic brain could be up-regulated by simvastatin [12], but the underlying mechanism had not been sufficiently investigated. Recently, Feng and co-workers reported that over-expression of endothelial nitric oxide synthase (eNOS) in cardiac tissues could increase SDF-1α expression in mice with ischemic-reperfusion injury [13]. As is well known that stabilization of eNOS mRNA and increase of nitric oxide (NO) production are the main mechanisms by which statins confer pleiotropic effects on cardiovascular diseases [14,15]. Taken together, it is reasonable to speculate that statins, the commonly used and easily available medication, may be the potential candidate used to enhance SDF-1α expression immediately after ischemic insult. Furthermore, we hypothesized that increase of SDF-1α might be, at least partially, attributable to eNOS upregulation and NO production by statins treatment, and the reciprocal coupling of SDF-1α with CXCR4 might be another novel mechanism by which statins confer its pleiotropic effects on cardiovascular diseases.

## Methods

### AMI production and atorvastatin administration

Healthy male Sprague–Dawley rats weighing 200-220g were obtained from the Experimental Animal Center of Sun Yat-sen University, Guangzhou, China. The study was approved by the Ethic Committee of Sun Yat-sen University. All animals received humane care in compliance with the Guide for the Care and Use of Laboratory Animals of the Institute of Laboratory Animal Resources, National Research Council.

Induction of AMI was performed as previously described by permanent ligation of the left anterior descending coronary artery [16]. And 36 survived rats were randomly and evenly assigned into four groups as blank control (B), atorvastatin (A), atorvastatin plus NG-Nitro-L-arginine Methyl Ester (L-NAME) (group A+L-NAME), and atorvastatin plus AMD3100 (group A+AMD3100). Another 9 rats underwent similar procedure but without coronary artery ligation were used as sham operated (S). Atorvastatin (10mg/Kg/d body weight, kindly offered by Pfizer Inc., solved in normal saline) was administrated by gavage once daily in the morning to group A, group A+L-NAME and group A+AMD3100, and same volume of saline was given to group B and group S. Eight hours later, L-NAME (40mg/Kg/d body weight, Sigma, St. Louis, MO , N5751) was given by gavage to group A+L-NAME, same volume saline was given to the other groups, and AMD-3100 (5mg/Kg/d body weight, Sigma, St. Louis, MO, A5602) was given to group A+AMD3100 by subcutaneous injection, same volume saline was subcutaneously injected to the other groups, the administration was once daily also. The general course was 7 days. One day after AMI, three rats from each group were used for cardiac function analysis, and then followed by heart excision for NO and SDF-1α evaluation. Seven days later, the rest rats were used for the following assessments after echocardiography was performed for cardiac function evaluation.

### Expression of eNOS /p-eNOS, STAT3 and CXCR4 in myocardium by Western blot

Seven days after AMI, expression of eNOS, p-eNOS, STAT3 and CXCR4 was detected by Western blot. Briefly, protein was extracted from infarcted and peri-infarcted regions of myocardium. Tissues were lysed with 500 μL of lysis buffer (50 mM Tris–HCl, pH 7.5; 5 mM EDTA; 250 mM NaCl; and 0.1% Triton X-100) containing 20μL (10mg/ml) of protease inhibitors Phenylmethanesulfonyl Fluoride (PMSF). Concentration of protein was measured by Bicinchoninic acid (BCA) method. Twenty micrograms of protein was electrophoresed on an 8% SDS-polyacrylamide gel and then transferred to a 0.22μm pore-sized Polyvinylidene Fluoride (PVDF) membrane. After blocking with 0.1% Tween in Tris-buffered saline (TBS-T) containing 5% BSA at room temperature for 1h, membranes were incubated at 4°C overnight with rabbit anti-rat eNOS, p-eNOS (Cell Signaling Technology, Danvers, MA, #9586 and #9570, 1:1000), rabbit anti-rat STAT3 (Cell Signaling Technology, Danvers, MA, #4904, 1:1000), and rabbit anti-rat CXCR4 (Santa Cruz, CA, sc-9046, 1:50). The membranes were washed three times with TBS-T and incubated with goat anti-rabbit IgG HRP-conjugated secondary antibody (Cell Signaling Technology, Danvers, MA, #7074, 1:3000) at room temperature for 1h. Interested protein was detected by chemiluminescence, and optical density (OD) was measured in grey scale images with Volume Contour method. GAPDH (Cell Signaling Technology, Danvers, MA, #2118, 1:6000) was used as a loading control. All the data were presented as relative expression after normalized to GAPDH. All the measurements were repeated for 3 times to figure out the arithmetic average.

### Evaluation of inflammatory cytokines and anti-apoptotic and pro-apoptotic proteins by Western blot

After seven days of AMI, expression of inflammatory cytokines (TGF-β, IL-1, IL-6, and TNF-α), pro-apoptotic (Bax) and anti-apoptotic (Bcl-2 and p-Akt) signals were detected by Western blot. Tissues were extracted from infarcted and peri-infarcted regions of myocardium, and then homogenized in 500 μL lysate (described aboved). After electrophoresis, transmembrane, and blocking, membrane was incubated at 4°C overnight with rabbit anti-rat TGF-β (Cell Signaling Technology, Danvers, MA, #3711, 1:1000), or IL-1, IL6 (Sigma, St. Louis, MO, I5018, SAB4300383, 1:1000), or TNF-α (Cell Signaling Technology, Danvers, MA, #3727, 1:1000), or Bax (Cell Signaling Technology, Danvers, MA, #2772, 1:1000), or Bcl-2 (Cell Signaling Technology, Danvers, MA, #2876, 1:1000) or p-Akt (Cell Signaling Technology, Danvers, MA, #9275, 1:1000). The membranes were washed three times with TBS-T and incubated with goat anti-rabbit IgG HRP-conjugated secondary antibody (Cell Signaling Technology, Danvers, MA, #7074, 1:3000) at room temperature for 1h. The rest procedures were as described above. All the measurements were repeated for 3 times to figure out the arithmetic average.

### Measurement of NO production in cardiac tissue

Total nitric oxide (NO) was evaluated by nitrite reductase method using Total Nitric Oxide Kit (Beyotime, Haimen, China, S0023). All the procedures were performed according to the manufacturer’s protocol. NO productions in the first and seventh day of AMI were evaluated. Tissue from the infarcted and peri-infarcted regions of myocardium was homogenized in lysate with 25 mmol/L Tris, 1% Triton X-100, 0.5 mmol/L EDTA, 150 mmol/L NaCl, 10 mmol/L NaF and a protease inhibitor PMSF 20μL (10mg/ml). After quantified by the Bicinchoninic acid (BCA) assay, protein concentration was adjusted to 10mg/ml. Potassium Nitrite (KNO_2_) supplied within the Total Nitric Oxide Kit was used to figure out standard curve. Optical density of each sample was detected by Enzyme-labeling measuring instrument at a wavelength of 550nm, and concentration was figured out on the standard curve. All the measurements were repeated for 3 times to figure out the arithmetic average.

### ELISA assay for SDF-1α expression

Quantitative immunoassay was used for evaluating expression of SDF-1α in the first and seventh day after AMI. Procedures were performed according to the manufacturer’s protocol (R&D Systems, Minneapolis, MN). Tissues from the infarcted and peri-infarcted regions of myocardium were homogenized in lysate (as described above). Protein concentration was adjusted to 10mg/ml. Recombinant murine SDF-1α supplied within the kit was used to figure out standard curve. Optical density of each sample was detected by Enzyme-labeling measuring instrument at a wavelength of 460nm, and the concentration was figured out on the standard curve. All the measurements were repeated for 3 times to figure out the arithmetic average.

### Hematoxylin-eosin staining and TUNEL detection

Cardiac tissues were embedded in 4μm paraffin sections for histological and TdT-mediated dUTP nick-end labeling (TUNEL) detections. The degree of inflammatory cells infiltration was evaluated by counting the number of inflammatory cells in 10 randomly peri-infarcted areas of each group at 200× magnification. Apoptotic index in peri-infarct areas was evaluated by TUNEL (Roche, Germany) 7 days after AMI. Procedures were performed according to the manufacture’s instruction. Slides were examined under a light microscope at 400× magnification. Normal cardiomyocytes nuclei were stained as blue while apoptotic ones were brown. Negative control (NC) was stained with removal of terminal transferase. The apoptotic index was calculated as TUNEL-positive nucleus divided by total number of nucleus in each field. Cells were counted at 10 random fields.

### Cardiac function analysis with echocardiography

Parameters of cardiac function were evaluated before, 1 and 7 days after AMI. Left ventricular ejection fraction (LVEF), fractional shortening (FS), left ventricular end-systolic and end-diastolic volume (LVVs and LVVd), heart rate (HR) and cardiac output (CO) were evaluated in each group with an echocardiography system (ATL-HDI500) equipped with a 10-MHz image transducer. Accordingly, CO was calculated with the following formula: CO=LVVd-LVVs. All procedures were performed by an experienced investigator who was blinded to the groups. All the measurements were repeated for 3 times.

### Statistical analysis

All continuous variables were expressed as mean±SD, and analyses were performed with SPSS software, version 16.0 (SPSS Science, Chicago, IL, USA). Statistical significance among different groups was evaluated with ANOVA post-hoc tests, and a value of *P* < 0.05 was considered to be statistically significant.

## Results

### Expression of eNOS and p-eNOS in myocardium was up-regulated by atorvastatin administration

**Figure 1:**
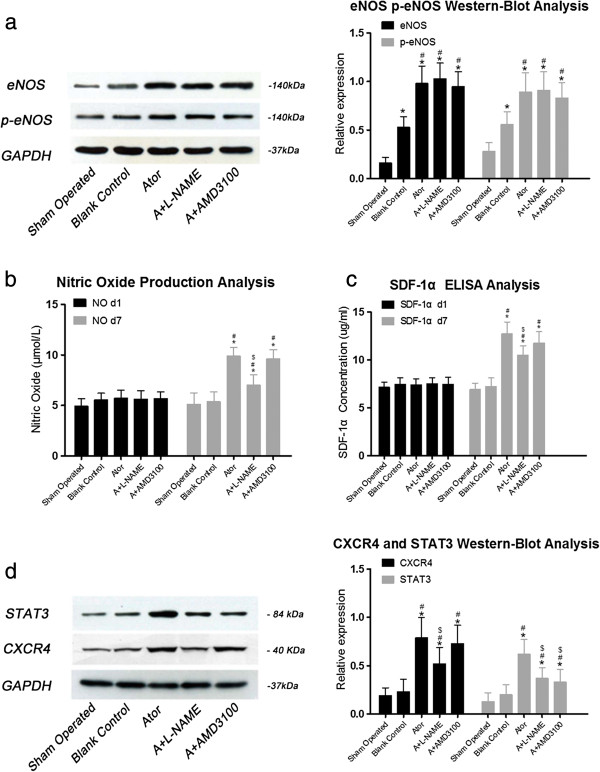
**Panel (a), 7 days after AMI, eNOS was significantly increased in AMI groups when compared with group S, and the three atorvastatin groups presented higher augmentation of eNOS.** (S: 0.16±0.06, B: 0.53±0.11*, A: 0.98±0.18*#, AL: 1.03±0.16*#, AA: 0.95±0.15*#). Similar result was observed in p-eNOS (S: 0.28±0.09, B: 0.56±0.13*, A: 0.89±0.20*#, AL: 0.91±0.83*#, AA: 0.83±0.16*#). (*: *P*<0.01 vs. group S, #: *P*<0.01 vs. group B). As shown in panel (**b**) and (**c**), one day after AMI, AMI groups showed slightly higher level of NO production than group S (S: 5.11±0.78, B: 5.56±0.66, A: 5.71±0.81, AL: 5.62±0.86, AA: 5.69±0.68, *P*=0.042 vs. group S). Similar trend was also observed in SDF-1α expression (S: 7.12±0.57, B: 7.43±0.71, A: 7.37±0.66, AL: 7.51±0.62, AA: 7.47±0.73, *P*=0.044 vs. group S). Seven days after AMI, NO production was significantly increased in three atorvastatin groups when compared with group B, while increment in group A+L-NAME was abrogated (S: 5.12±1.10, B: 5.36±0.98, A: 9.89±0.86*#, AL: 7.01±1.03*#$, AA: 9.60±0.92*#). SDF-1α expression in three atorvastatin groups was also increased when compared with group B, while the increment was reduced in group A+L-NAME after NO production was diminished by L-NAME (S: 6.91±0.67, B: 7.22±0.89, A: 12.73±1.23*#, AL:10.51±0.95*#$, AA:11.59±1.16*#). (*: *P*<0.01 vs. group S, #: *P*<0.01 vs. group B, $: *P*<0.01 vs. group A and group AA). As shown in panel (**d**), 7 days after AMI, CXCR4 was significantly increased in all three atorvastatin groups. (S: 0.19±0.08, B: 0.23±0.13, A: 0.79±0.21*#, AL: 0.52±0.17*#$, AA: 0.73±0.19*#). Similar result was observed in STAT3 (S: 0.13±0.09, B: 0.20±0.10, A: 0.62±0.15*#, AL: 0.37±0.11*#$, AA: 0.33±0.13*#$). (*: *P*<0.01 vs. group S, #: *P*<0.01 vs. group B, $: *P*<0.01 vs. group A) All the measurements were repeated for 3 times to figure out arithmetic average. S=Sham operated group; B=Blank control group; A=Atorvastatin group; AL=Atorvastatin+L-NAME group; AA=Atorvastatin+AMD3100 group.

As shown in Figure 1 (panel a), seven days after AMI, comparing with group B, eNOS and p-eNOS were significantly increased in all three atorvastatin therapy groups (*P*<0.05) and the increment among these groups were comparable, indicating that short term of atorvastatin treatment had robust effects on eNOS and p-eNOS upregulation.

### Increases of NO production and SDF-1α expression in myocardium by atorvastatin treatment

As shown in Figure 1 (panel b), one day after AMI, NO production in all AMI groups were slightly increased when compared with group S. Seven days later, NO production in both group A and group A+AMD3100 was significantly increased (*P*<0.05, vs group B), while the efficacy of atorvastatin on NO production was abrogated by L-NAME addition, as evidenced by the NO production in group A+L-NAME was reduced. SDF-1α concentration was concomitantly evaluated by ELISA. As shown in Figure 1 (panel c), one day after AMI, comparing with group S, SDF-1α expression in all AMI groups were also slightly increased, consistent with previous report that SDF-1α could spontaneously increase after ischemic insult. Seven days later, SDF-1α expression in both group A and group A+AMD3100 was profoundly up-regulated when compared with group A+L-NAME, indicating that inhibition of NO production diminished SDF-1α expression. Additionally, SDF-1α expression in group B in day 7 was similar to that in day 1, indicating that spontaneous upregulation of SDF-1α after ischemic insult was a transient and temporal course.

### Expression of STAT3 and CXCR4 in myocardium was up-regulated by atorvastatin treatment

As shown in Figure 1 (panel d), seven days after AMI, comparing with group B, CXCR4 and STAT3 expressions were significantly increased in all three atorvastatin therapy groups (*P*<0.05). The expression of CXCR4 in group A and group A+AMD3100 were significantly higher than in group A+L-NAME, indicating that the elevation of CXCR4 in cardiac tissues was also related to eNOS/NO pathway. Although the expressions of CXCR4 and SDF-1α in group A and group A+AMD3100 were comparable, the expression of STAT3 in group A+AMD3100 was significantly lower than in group A, supporting the notion that STAT3 expression was mediated by CXCR4 and SDF-1α coupling.

### Anti-inflammatory effects of atorvastatin

**Figure 2:**
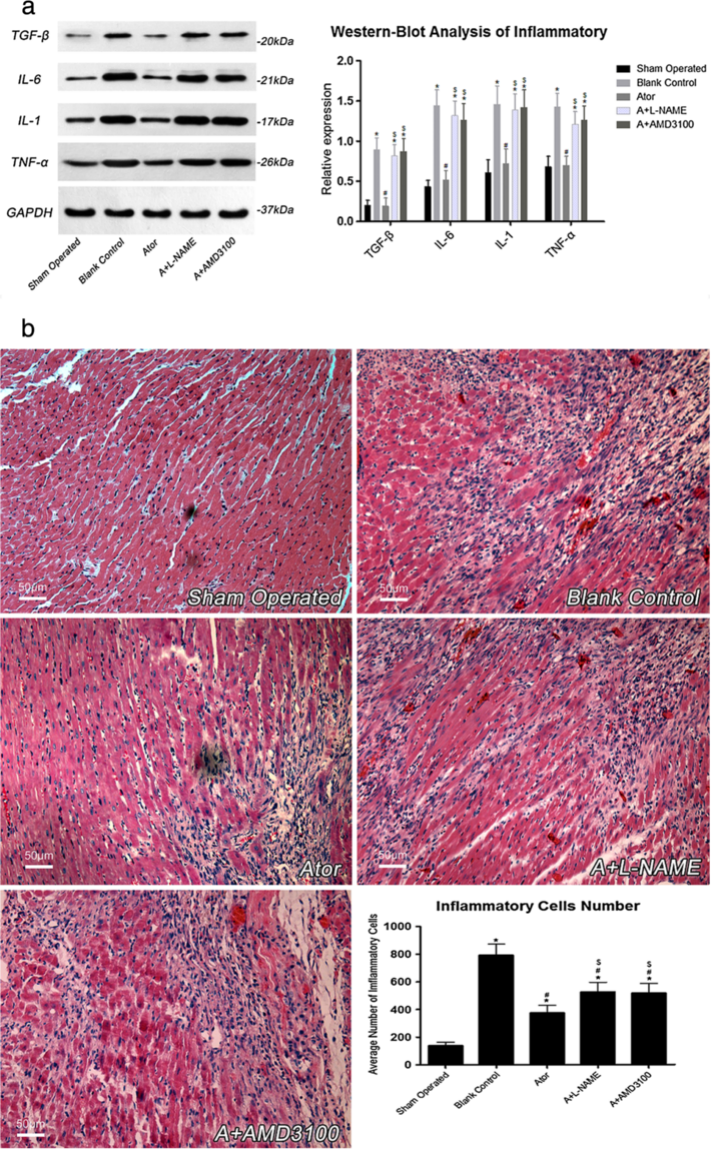
**Panel (a) and (b), 7 days after AMI, inflammatory cytokines expression atorvastatin treated group was significantly diminished when compared with group B.** Meanwhile, the number of inflammatory cells infiltration was also concomitantly reduced in atorvastatin treated groups. And the effects of anti-inflammation by atorvastatin treatment were abrogated by L-NAME or AMD3100, as demonstrated by that both the expressions of inflammatory cytokines and the number of inflammatory cells were significantly reduced in group A than group A+L-NAME and group A+AMD3100. TGF-β (S: 0.18±0.06, B: 0.89±0.15*, A: 0.26±0.09*#, AL: 0.82±0.10*$, AA: 0.87±0.16*$), IL-6 (S: 0.40±0.08, B: 1.44±0.20*, A: 0.58±0.11*#, AL: 1.32±0.18*$, AA: 1.26±0.21*$), IL-1 (S: 0.56±0.13, B: 1.46±0.23*, A: 0.75±0.18*#, AL: 1.39±0.20*$, AA: 1.42±0.22*$), TNF-α (S: 0.63±0.10, B: 1.43±0.17*, A: 0.77±0.11*#, AL: 1.21±0.16*$, AA: 1.26±0.18*$). Inflammatory cells number (S: 137.2±26.4, B: 792.1±81.7*, A: 375.3±55.4*#, AL: 527.5±69.1*#$, AA: 516.3±71.4*#$). (*: P<0.01 vs. group S, #: P<0.01 vs. group B, $: P<0.01 vs. group A). All the measurements were repeated for 3 times to figure out the arithmetic average. S=Sham operated group; B=Blank control group; A=Atorvastatin group; AL=Atorvastatin+L-NAME group; AA=Atorvastatin+AMD3100 group.

As shown in Figure 2 (panel a and b), seven days after AMI, inflammatory reaction was mitigated in atorvastatin treatment group when compared with group B. Inhibition of NO production with L-NAME or blockage of SDF-1α with CXCR4 coupling by AMD3100 could potently offset the anti-inflammatory effects of atorvastatin, as evidenced by the expression of inflammatory cytokines and the number of inflammatory cells in group A+L-NAME and group A+AMD3100 were higher than in group A (*P*<0.05).

### Anti-apoptotic effects of atorvastatin

**Figure 3:**
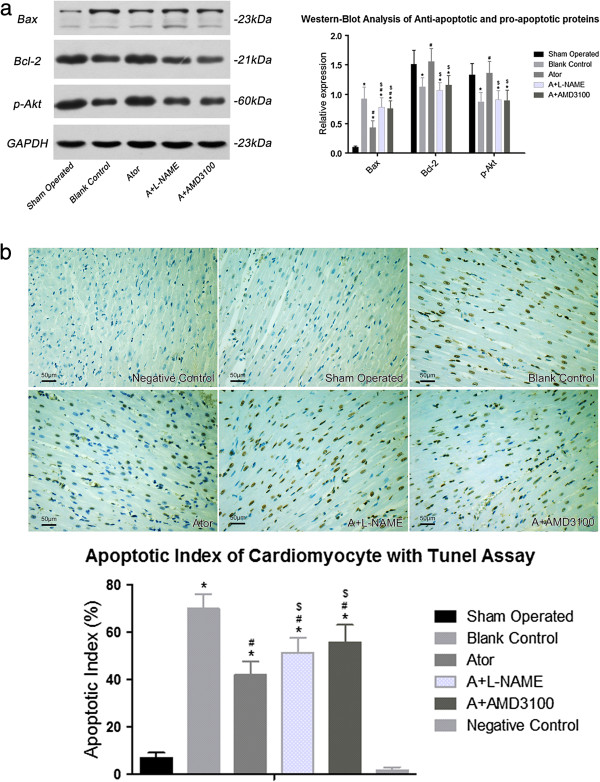
**Panel (a) and (b), 7 days after AMI, Western blot analysis revealed that atorvastatin treatment increased the pro-survival proteins and reduced the pro-apoptotic protein, however, this effects was diminished by either L-NAME or AMD3100.** Bcl-2 (S: 1.51±0.24, B: 1.13±0.15*, A: 1.56±0.22#, AL: 1.07±0.13*$, AA: 1.16±0.16*$) and p-Akt (S: 1.33±0.19, B: 0.87±0.16*, A: 1.36±0.20#, AL: 0.91±0.15*$, AA: 0.89±0.18*$), and Bax (S: 0.10±0.02, B: 0.92±0.20*, A: 0.43±0.12*#, AL: 0.78±0.16*#$, AA: 0.76±0.13*#$). Apoptotic index of cardiomyocytes by TUNEL assay further suggested that the anti-apoptotic effects of atorvastatin was associated with increase of NO production and SDF-1α expression, and the coupling of SDF-1α with CXCR-4 (S: 6.94±2.12, B: 70.09±5.99*, A: 42.12±5.49*#, AL: 58.37±6.28*#$, AA: 55.72±7.31*#$). (*: *P*<0.01 vs. group S, #: *P*<0.01 vs. group B, $: *P*<0.01 vs. group A). All the measurements were repeated for 3 times to figure out the arithmetic average. S=Sham operated group; B=Blank control group; A=Atorvastatin group; AL=Atorvastatin+L-NAME group; AA=Atorvastatin+AMD3100 group.

Western blot and TUNEL were performed to evaluate the anti-apoptotic effects of atorvastatin. As shown in Figure 3 (panel a and b), both anti-apoptotic proteins Bcl-2 and p-Akt were significantly up-regulated, accompanying with significant down-regulation of pro-apoptotic protein Bax and reduction of apoptotic index, in all three atorvastatin treated groups when compared with group B. Compared with group A, the effects of atorvastatin on anti-apoptosis in group A+L-NAME and group A+AMD3100 were diminished, indicating that upregulation of NO and SDF-1α, and coupling of SDF-1α with CXCR4 were the underlying mechanisms by which atorvastatin conferred anti-apoptotic effects.

### Cardiac function was improved by 7 day-atorvastatin therapy

Parameters of cardiac function were listed in Table 1. Briefly, one day after AMI, significant impairment of LVEF, FS and CO was detected in all AMI groups comparing with group S. Enlargement of LVVs and LVVd in all AMI groups also showed an acute dysfunction of left ventricle. There was no statistical difference of cardiac function among these four AMI groups on day 1 after AMI. After 7 days of atorvastatin therapy, LVEF, FS and CO were significantly improved, accompanying with reduction of LVVs, LVVd and HR in group A, group A+L-NAME and group A+AMD3100 when compared with group B. However, the improvement of cardiac function was less prominent in group A+L-NAME and group A+AMD3100 than in group A, indicating that the protective effect on cardiac function with atorvastatin treatment was associated with the elevation of NO and SDF-1α, and the coupling of SDF-1α with CXCR4.

**Table 1:** Cardiac function analysis with echocardiography

**Groups**	**S**	**B**	**A**	**AL**	**AA**
**Before AMI**					
**LVEF(%)**	70.52±2.09	69.27±2.33	68.75±3.01	70.11±2.67	69.35±2.82
**FS(%)**	35.98±3.15	34.83±4.02	37.09±3.73	36.62±4.09	35.26±3.35
**LVVs(ml)**	2.03±0.27	1.99±0.35	2.16±0.31	2.08±0.30	2.05±0.28
**LVVd(ml)**	5.76±0.30	5.97±0.38	5.85±0.41	5.81±0.34	5.85±0.31
**CO(ml)**	3.72±0.18	3.98±0.27	3.69±0.22	3.73±0.21	3.81±0.25
**HR(bpm)**	454±68	462±59	455±61	466±78	459±71
**One day after AMI**					
**LVEF(%)**	69.51±1.94	46.13±2.61*	45.26±2.44*	46.09±2.72*	45.68±2.59*
**FS(%)**	35.78±3.23	25.71±3.06*	26.07±2.99*	25.22±3.30*	24.97±3.26*
**LVVs(ml)**	2.08±0.31	3.52±0.31*	3.47±0.36*	3.56±0.29*	3.45±0.30*
**LVVd(ml)**	5.82±0.27	6.49±0.37*	6.71±0.35*	6.55±0.42*	6.61±0.39*
**CO(ml)**	3.74±0.19	2.97±0.22*	3.24±0.27*	2.99±0.24*	3.16±0.25*
**HR(bpm)**	461±70	536±67	551±56	549±62	541±71
**Seven days after AMI**					
**LVEF(%)**	70.07±2.11	44.91±2.72*	55.57±3.61*#	49.98±3.03*#$	48.98±2.87*#$
**FS(%)**	36.09±3.12	23.96±3.41*	32.58±3.35#	27.20±3.27*#$	27.86±3.32*#$
**LVVs(ml)**	2.04±0.25	3.63±0.37*	2.71±0.53*#	3.11±0.46*#	3.18±0.41*#
**LVVd(ml)**	5.67±0.23	6.53±0.29*	6.03±0.28*#	6.26±0.24*	6.41±0.26*$
**CO(ml)**	3.63±0.21	2.90±0.24*	3.32±0.31	3.09±0.26*	3.23±0.29*#$
**HR(bpm)**	457±63	547±66	488±57	517±68	522±63

· * P<0.05 vs. Sham group.

· # P<0.05 vs. Blank Control group.

· $ P<0.05 vs. Atorvastatin group.

· S=Sham operated group; B=Blank control group; A=Atorvastatin group; AL=Atorvastatin+L-NAME group; AA=Atorvastatin+AMD3100 group.

## Discussion

Our present study showed that short term of atorvastatin treatment could potently augment SDF-1α and CXCR4 expression in ischemic cardiac tissue, which was associated with the upregulation of eNOS/p-eNOS and increase of NO. Furthermore, STAT3, which is a cardioprotective factor, was also significantly increased through SDF-1α and CXCR4 coupling. SDF-1α upregulation and SDF-1α coupling with its cognate receptor CXCR4 contributed to the pleiotropic effects of atorvastatin in terms of anti-inflammation and anti-apoptosis. Our study presented a new insight into the roles and mechanisms of atorvastatin treatment on SDF-1α and CXCR4 expression, and upregulation of SDF-1α and CXCR4 might be one of the mechanisms by which atorvastatin confer its pleiotropic effects. With regard to the “off the shelf” advantage, we suggested that atorvastatin might be a competent candidate used to immediately up-regulate SDF-1α expression after urgent ischemic insult, rather than intricately genetic approaches.

Statins are a class of medication which have been broadly used in prevention and treatment of cardiovascular diseases. Other than the potent efficacy on lipid profiles regulation, statins have pleiotropic effects in terms of anti-inflammation, anti-oxidation, anti-apoptosis, stem cells mobilization and plaque stabilization [17-20]. One of the important mechanisms contributing to these pleiotropic effects is the stabilization of eNOS mRNA and increase of eNOS expression [15,21]. NO is generated by eNOS and modulates multiple processes contributing to improving cardiac function after ischemic insult [22,23]. Recently, one study showed that eNOS over-expression in cardiac tissues by genetic approach could up-regulate SDF-1α expression [13]. However, whether SDF-1α could be up-regulated by statins via eNOS/NO dependent pathway and whether SDF-1α is the novel mechanism by which statins confer its pleiotropic effects on cardiovascular diseases are still unclear. In our present study, we showed that after seven days of atorvastatin treatment, SDF-1α expression was significantly increased in three atorvastatin treated groups, accompanying with its specific receptor CXCR4. But this efficacy was significantly abrogated when NO production was diminished by L-NAME administration in group A+L-NAME when compared with group A and group A+AMD3100, indicating that SDF-1α and CXCR4 upregulation were largely attributed to NO production, which, to our best knowledge, was the first time to reveal that short term of atorvastatin treatment could significantly increase SDF-1α and CXCR4 expression in ischemic cardiac tissues via the eNOS/NO dependent pathway. However, the precise mechanism by which NO up-regulate SDF-1α and CXCR4 expression is still unclear. In Feng’s study [13], they found that in the ischemic/reperfusion injury model, the increase of SDF-1α at least partially via activation of soluble guanylyl cyclase and increase of cGMP. In our study, the rats were underwent permanent ligation of coronary artery which was different from Feng’s study, and data from our pilot project showed that neither adenlyly cyclase nor guanylyl cyclase signaling pathway resulted in convincing results. Therefore, the exact mechanism of SDF-1α up-regulation remained inconclusive. Additionally, although NO production in group A+L-NAME was abrogated at the seventh day, the SDF-1α expression in group A+L-NAME was still significantly higher than group B, indicating that SDF-1α upregulation might also be ascribed to other mechanisms.

As it is well known that atorvastatin has anti-inflammatory and anti-apoptotic effects. The mechanisms are ascribed to the protective effects on endothelial cells, production of NO, dilatation of vessel, activation of pro-survival and inactivation of pro-apoptotic pathway. And in Cui’s study [12], they revealed that simvastatin treatment could up-regulate SDF-1α expression in ischemic brain which in turn to increase number of transplanted stem cells to lesion area, and facilitate neo-vascularization and improve the outcome. However, the effects and mechanisms of SDF-1α coupling with CXCR4 in ischemic brain tissues had not been intensively investigated in Cui’s study. Literally, both SDF-1α and CXCR4 simultaneously express in cardiac tissues, and the beneficial effects of the pathway confers in ischemic cardiac diseases have been intensively investigated, mostly with genetic approach. In our present study, we revealed that SDF-1α and CXCR4 expression, under the AMI setting, could be up-regulated by short term of atorvastatin treatment. Furthermore, the upregulation of SDF-1α and CXCR4 could render robust anti-inflammatory and anti-apoptotic effects via the coupling of SDF-1α with CXCR4, as evidenced by that the efficacies were offset either when AMD3100, the specific antagonist of CXCR4, was administrated in group A+AMD3100, or the expressions of SDF-1α and CXCR4 were reduced in group A+L-NAME.

Finally, our present study showed that the protective effects on cardiac function by atorvastatin treatment was associated with the upregulation of SDF-1α and the coupling of SDF-1α with CXCR4, which was demonstrated by cardiac function in terms of LVEF, FS and CO, was significantly reduced when L-NAME or AMD3100 was administrated. Moreover, since the expression of STAT3, a critical cardioprotective factor that mediates SDF-1α/CXCR4 axis induced cardiac protection, was up-regulated most significantly in group A than the other groups, we considered that STAT3 upregulation owing to SDF-1α and CXCR4 coupling might partially responsible for cardiac function improvement.

## Conclusion

With regard to the intensive advocates “from bench to bedside” and “bridging basic research to clinical benefit” nowadays, our present study for the first time identified a new hint, which we consider to be a simple and clinically feasible approach, to up-regulate SDF-1α expression by commonly used medication statins rather than by previously complicated genetic approaches. Furthermore, with regard to the effects on mobilization and engraftment of stem cells by SDF-1α and CXCR4 axis, upregulation of SDF-1α in ischemic cardiac tissues by statins therapy could be a promising way in terms of myocardial regeneration in the future.

## Competing interests

All authors declare that there is no conflict interest.

## Authors’ contribution

RQ and AC wrote this manuscript, RQ, AC, YH and DZ participated in this research, YD, YZ and DY assisted in research carried out, SR carried out statistical analysis, YF and WM assisted in manuscript revision. All authors read and approved the final manuscript.

## Authors’ information

Ruofeng Qiu and Anping Cai co-first authors.

## Funding

This work was supported by the grants from the Technology Project Foundation of Guangdong Province, China (2009A030301004, 2011B031800021 and 2011B031800263). The research grant of cardiovascular medication of Guangdong Province (2011X25). Medical Scientific Research Grant of the Health Ministry of Guangdong province, China (B2011310, A2012663).
